# Innovative rehabilitative bracing with applied resistance improves walking pattern recovery in the early stages of rehabilitation after ACL reconstruction: a preliminary investigation

**DOI:** 10.1186/s12891-020-03661-z

**Published:** 2020-10-02

**Authors:** Jacopo Emanuele Rocchi, Luciana Labanca, Valeria Luongo, Lorenzo Rum

**Affiliations:** 1 Department of Movement, Human and Health Sciences, University of Rome “Foro Italico”, Piazza Lauro De Bosis 6, 00135 Rome, Italy; 2 Villa Stuart Sport Clinic, FIFA Medical Centre of Excellence, Via Trionfale 5952, 00135 Rome, Italy

**Keywords:** Anterior cruciate ligament, Brace, Rehabilitation, Gait, Biomechanics

## Abstract

**Background:**

The use of knee braces early after anterior cruciate ligament (ACL) reconstruction is a controversial issue. The study preliminarily compares the effect of a traditional brace blocked in knee extension and a new functional brace equipped with a spring resistance on walking and strength performance early after ACL reconstruction performed in the acute/subacute stage.

**Methods:**

14 ACL-reconstructed patients wore either a traditional (Control group: CG, 7 subjects) or a new functional brace (Experimental group: EG 7 subjects) until the 30^th^ post-operative day. All patients were tested before surgery (T0), 15, 30, and 60 days after surgery (T1, T2, and T3, respectively). Knee angular displacement and ground reaction forces (GRF) during the stance phase of the gait cycle were analyzed at each session and, at T3, maximal voluntary isometric contraction (MVIC) for knee flexor/extensor muscles was performed. Limb symmetry indexes (LSI) of GRF and MVIC parameters were calculated.

**Results:**

At T3, EG showed greater peak knee flexion angle of injured limb compared to CG (41 ± 2° *vs* 32 ± 1°, *p* < 0.001). During weight acceptance, a significant increase of anteroposterior GRF peak and vertical impulse from T1 to T3 was observed in the injured limb in EG (*p* < 0.05) but not in CG (*p* > 0.05). EG showed a greater side-to-side LSI of weight acceptance peak of anteroposterior GRF at T2 (113 ± 23% *vs* 69 ± 11%, *p* < 0.05) and T3 (112 ± 23% *vs* 84 ± 10%, *p* < 0.05).

**Conclusions:**

The preliminary findings from this study indicate that the new functional brace did help in improving gait biomechanical pattern in the first two months after ACL reconstruction compared to a traditional brace locked in knee extension.

## Background

Anterior cruciate ligament (ACL) tear is one of the most common injuries in pivoting and cutting sports that very often ends up with ligament reconstruction [1, 2]. Therefore, the development of effective post-surgical rehabilitation protocols capable of mitigating the future risk of knee-related issues plays an essential role in the safe return to previous activities, i.e. sport practice and/or activities of daily living [3–6]. Although being standardized in most of the clinical settings, the rehabilitation protocol should be susceptible to changes and adapted to the needs of the single patient at different timings over the entire recovery process [7]. Early post-surgical ACL rehabilitation (0–6 weeks post-surgery) usually involves the loading and mobilization of the injured limb as well as the adoption of orthopedic braces. Despite its large adoption following ACL reconstruction [8], evidence from systematic reviews and meta-analysis indicates that traditional bracing adds no significant benefit in the short term on the clinical outcomes of function and stability when added to standard therapy [9–11]. Consequently, the adoption or not of a post-operative brace remains a subject of debate.

After ACL rupture, patients develop peculiar strategies of neuromuscular activation to prevent joint instability according to the motor task performed [12, 13]. In the mid- and long-term perspective, this is often associated with persistent thigh muscles weakness in the operated limb, thereby leading to a side-to-side asymmetry that alters normal knee and lower limb mechanics during daily motion [14, 15]. For instance, during walking and functional tasks, reductions in the peak of knee flexion angle, external knee flexion moment, vertical impulse as well as posterior peak of ground reaction force have been observed in the operated limb of patients with ACL lesion and reconstruction [16–19]. Accordingly, after the reconstruction, it is well known that reaching a complete range of motion is one of the main goals of the early rehabilitation [4]. Moreover, recent improvements in surgical practices have allowed to achieve a greater knee joint stability immediately after surgery [20], making more stressful rehabilitation procedures possible. In the current literature, the traditionally investigated types of knee brace include prophylactic, rehabilitative and functional braces [10], none of which directly applies a resistance to the flexion of knee joint over the entire range of motion. There is some evidence that traditional knee braces, especially functional ones, are able to improve the management of anteroposterior knee shear forces and increase knee flexion angle during walking in ACL-reconstructed patients [21–23]. Nevertheless, it is still not clear whether braces designed to resist/assist knee joint movement can actually improve the early phase of rehabilitation compared to generally adopted brace types. In particular, to which extent the neuromuscular alterations in flexors/extensors activation profiles and their related biomechanical outcomes after ACL reconstruction could be modified by a brace which facilitates knee extension and resists to flexion is yet to be investigated. Therefore, the study aimed at retrospectively evaluating the walking performance outcomes after the adoption of an innovative brace model in comparison to a traditional brace locked in full extension in ACL reconstructed patients during the early stages of the rehabilitation process. It was hypothesized that the proposed brace model would lead to a greater recovery of both gait mechanics and hamstrings force production in the first two months after surgery.

## Methods

### Study population

In this retrospective study, patient data was selected from the database of Villa Stuart Sport Clinic. All patient underwent ACL reconstruction in the period of April–May 2018. Before the study was initiated, approval from the institutional review board of the University of Rome “Foro Italico” was obtained to analyze the patients’ data. An apriori power analysis (G*Power 3.1.9.2 software) was calculated with a significance level of α = 0.05, a power of 90% and an effect size f of 0.30, with results indicating 22 subjects per group. However, only fourteen consecutive patients with unilateral isolated rupture of ACL who underwent surgical reconstruction from the database were eligible to be included in this study, which, therefore, has to be considered as preliminary. Criteria for including patients in the analysis were a complete active knee range of motion and absence of joint locking and knee swelling. Patients were excluded if a concomitant injury of any other ligament of both lower limbs, meniscal injury or previous surgical interventions on both knees were reported during the pre-surgery medical examination. Furthermore, patients who missed one or more follow-up testing sessions were excluded. Included patients were participating in recreational activities, with a physical activity level defined as a Tegner Activity Scale level 3–6 [24].

### Surgical procedure and rehabilitation protocol

**Fig. 1:**
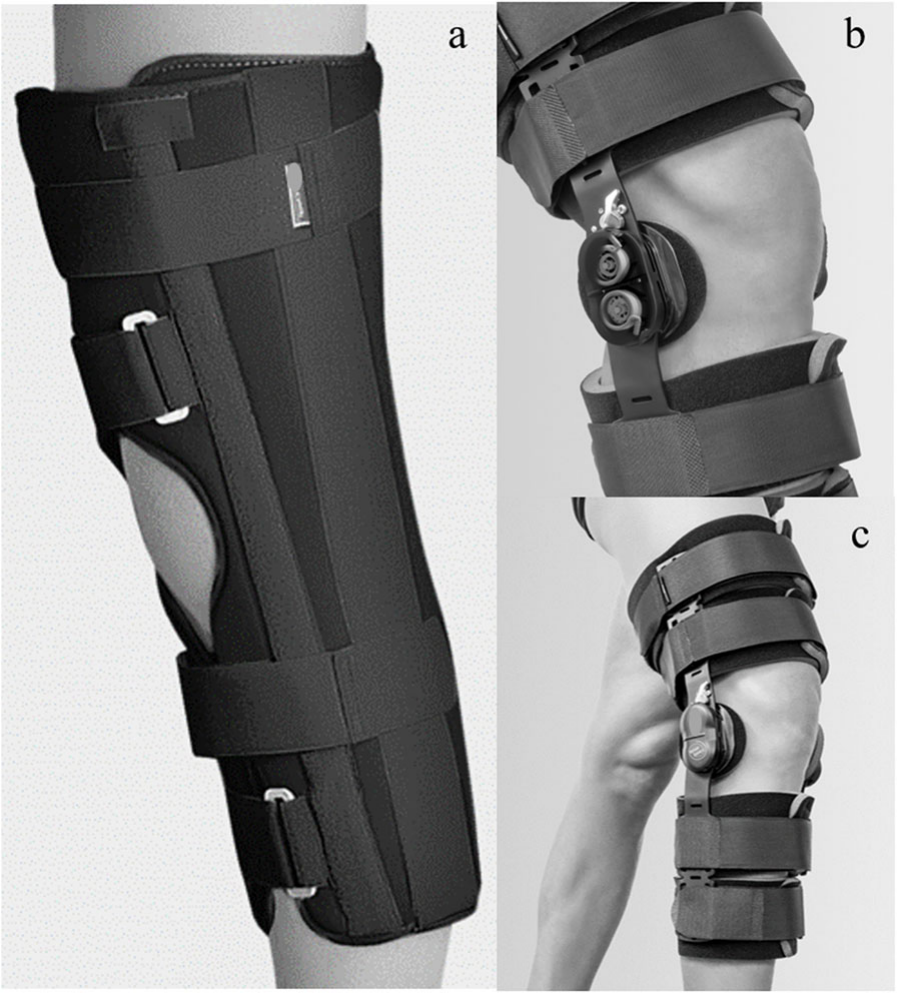
Representative picture of the two models of brace: traditional brace locked at 0° knee extension (a) and functional brace with resistance to knee flexion and its polycentric spring system (b and c). The picture was taken from the producing company’s website and permission to publish was obtained from the company

Surgical procedures included arthroscopic anatomical transtibial reconstruction of ACL with autologous transplantation from single-bundle patellar (PT, 7 subjects) or double-bundle semitendinosus and gracilis tendon (ST-GR, 7 subjects), performed by the same highly experienced surgeon between 10 and 30 days after the injury. After surgery, all patients wore a brace until the 30^th^ post-operative day and were granted full load on the operated limb from the 3^rd^ day. Two different types of brace were assigned at surgeon and physiatrist’s discretion. Seven subjects (control group, CG, 5 males and 2 females, 3 PT and 4 ST-GR, age: 37 ± 15 years; body mass: 61 ± 7 kg, height: 169 ± 9 cm) wore a traditional brace locked at 0° knee extension (GNT-601, FGP S.R.L., Dossobuono, Italy), while 7 subjects (experimental group, EG, 3 males and 4 females, 4 PT and 3 ST-GR, age: 34 ± 11 years; body mass: 77 ± 12 kg, height: 174 ± 1 cm) wore an innovative brace (RehaBrace, FGP S.R.L., Dossobuono, Italy) (Fig. 1).

This brace, in addition to the knee full-extension locking, is equipped with a polycentric junction spring system that creates a 2.5 kg resistance to knee flexion, thus promoting the activity of flexor muscles during the gait cycle with a complete range of motion. During the first 15 days after surgery, patients of both EG and CG had the brace locked in full knee extension, while the functional brace was unlocked to complete range of motion between 15 and 30 days after surgery in the EG. A standardized physiotherapy treatment of 5 consecutive days per week was administered to each patient until the end of the 2^nd^ month after surgery (Table 1).

**Table 1:** Rehabilitation protocol followed by ACL-reconstructed subjects from 15^th^ day to 2^nd^ month after surgery. NMES = Neuromuscular Electrical Stimulation; ROM = Range of Motion; CKC = Closed Kinetic Chain

1^st^ and 2^nd^ week
Weight bearing with brace
Passive mobilization
Active quadriceps NMES
Straight leg raises
Calf strengthening
Hamstrings light stretching
**2**^**nd**^** to 4**^**th**^** week**
Weight bearing with brace
Active mobilization
Active quadriceps NMES
Squatting exercises
Straight leg raises
Calf strengthening
Adductor strengthening
Hamstrings light stretching
**4**^**th**^** to 8**^**th**^** week**
Full ROM recovery
Weight bearing without brace
Active mobilization
Squatting exercises
Calf strengthening
Active quadriceps NMES
CKC resistance training
Quadriceps stretching
Hamstrings stretching

After obtaining consent to the use of personal data for research purposes from all patients, performance data was processed anonymously and in compliance with privacy.

### Experimental procedures and equipment

After a 10-min warm-up with a cycle ergometer, patients were asked to walk in a straight line at self-selected comfortable speed on an 8 m walkway. A force platform (model 9281 B; KISTLER, Winterthur, Switzerland) was embedded into the floor in the middle of the walkway to measure ground reaction forces (GRF) during the stance phase of gait cycle. A series of 5 to 10 familiarization trials depending on the degree of confidence were performed, to allow the subject to step onto the force plate with no changes in the walking behavior. Three trials per each leg (healthy and injured) were then performed in a randomized order. An electrogoniometer (Biometrics Ltd, Gwent, United Kingdom) was placed on the knee of the investigated lower limb with the two sensors aligned with thigh and shank axes. As part of the clinical routine, all patients underwent a total of 4 testing sessions at different timings: before the surgery (T0), at 15, 30 and 60 days after the surgery (T1, T2, and T3, respectively). At T3 subjects were tested for maximal voluntary isometric contraction (MVIC) of knee extensor muscles at 30° and 90° of knee flexion and of knee flexors at 90° of knee flexion in both limbs. During the assessment, patients were seated on a leg extension machine (Technogym, Forli-Cesena, Italy) for the knee extension and on a leg curl machine (Technogym) for the knee flexion contraction. Patients were fastened using three crossing belts on both machines. Muscle force was recorded using a load cell connected to a computerized system unit (MuscleLab; Bosco-System Technologies, Rieti, Italy). The maximal voluntary isometric contraction task consisted of a progressive increase to a maximum force exerted by the leg muscles. Patients were able to follow their performance on a computer screen and were verbally encouraged to achieve a maximum and to maintain that maximum for at least 2 s before relaxing. Maximal isometric force production was calculated as the higher 1-s average reached within the trials. Peak forces exerted by each limb were recorded.

### Data analysis

**Fig. 2:**
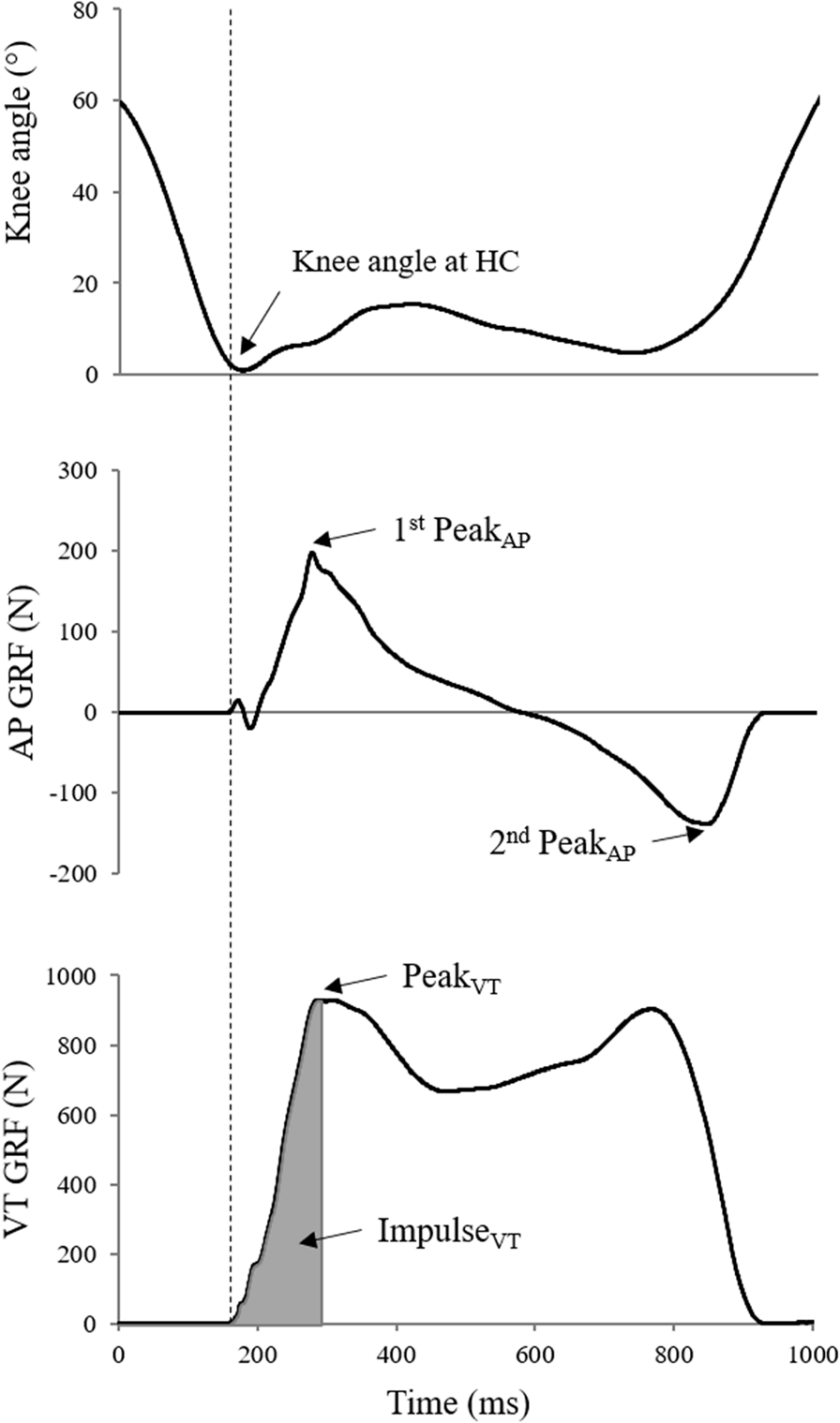
Knee angle, AP GRF and VT GRF of a representative trial. Vertical dotted line indicates the heel contact with the force plate. HC: heel contact; 1^st^ Peak_AP_: positive peak of AP GRF during weight acceptance; 2^nd^ Peak_AP_: negative peak of AP GRF during push-off; Peak_VT_: peak of VT GRF after heel contact; Impulse_VT_: impulse of VT GRF from heel contact to first peak

To evaluate the kinematic changes in the knee joint mobility during gait, the maximum flexion angle during the stance phase and the knee angle at heel contact with the force plate were calculated. The timing of heel contact with the force platform was defined as the instant at which the vertical component of GRF reached 10 N. The analysis of anteroposterior (AP) and vertical (VT) components of GRF was performed to investigate their management throughout the entire rehabilitation process. In particular, the first peak after heel contact (Peak_VT_) and the impulse from heel contact to the peak (Impulse_VT_) were computed from the VT GRF, while the two consecutive force peaks (1^st^ and 2^nd^ Peak_AP_) during the weight acceptance and push-off periods of stance phase, respectively, were obtained from the AP component (Fig. 2).

Maximal isometric force production was calculated as the higher 1-s average reached within the trials. Peak forces exerted by each limb were recorded at 30° (Ext_30_) and at 90° (Ext_90_) of knee flexion for extensors and at 90° of flexion for flexors (Flex_90_).

Each kinetic and force parameter was normalized by the subject’s body weight and was calculated for both the healthy and injured leg.

In addition, a limb symmetry index (LSI) for Peak_VT_, 1^st^ and 2^nd^ Peak_AP,_ Ext_30,_ Ext _90_ and Flex_90_ was calculated as follows:

$$ LSI=\frac{injured}{healthy}\times 100 $$

where the value of 100% indicates an equal capacity across the injured and healthy legs.

### Statistical analysis

An operator who was unaware of the group’s brace adoption performed all statistical analysis. A mixed-design analysis of variance (ANOVA) was conducted on both kinematic and kinetic parameters to evaluate the main effects of time (T0, T1, T2 and T3) and group (CG x EG), and their interaction (time x group). The assumptions of normal data distribution and equality of variance were evaluated using the Shapiro–Wilk test and the Levene’s test, respectively. When a significant interaction effect was observed, follow-up analysis with pairwise comparisons was separately performed within each group to examine differences across time measures and within each time measure for between-group differences. Post hoc procedures were also adopted for between- and within-group differences when significant main effects of group and time were found, respectively. One-way ANOVA was used to test group differences in LSI of MVICs at T3. Statistical analysis was conducted using SPSS (version 21.0, IBM, Chicago, IL). A significance level of α was set at 0.05 and further follow-up analyses are provided with the Bonferroni corrected *p*-values.

## Results

**Fig. 3:**
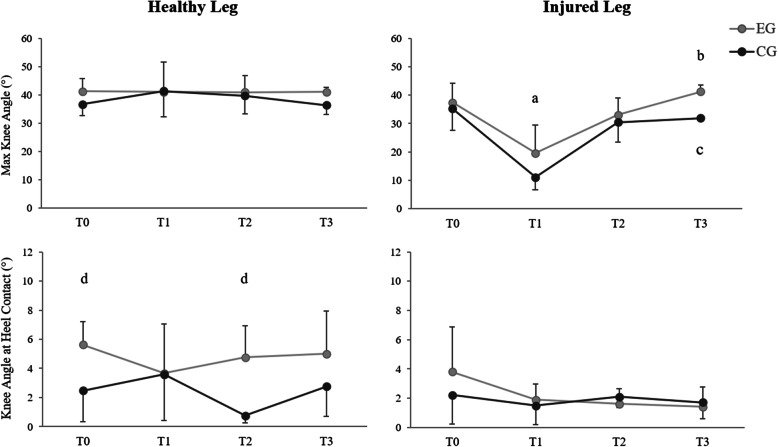
Mean and standard deviation of maximum knee flexion angle and knee flexion angle at heel contact in the healthy and injured leg. ^a^ Significantly different *vs* T0, T2 and T3 in both EG and CG (*p* < 0.05); ^b^ Significantly different *vs* T2 in EG (*p* < 0.05); ^c^ Significantly different EG *vs* CG (*p* < 0.001); ^d^ Significantly different *vs* CG (*p* < 0.05)

Significant main effects of time (F(3,36) = 40.143, *p* < 0.001) and group (F(1,12) = 8.524, *p* < 0.05) were found in maximum knee flexion angle of the injured leg (Fig. 3). Post hoc analysis revealed that angle values at T1 were significantly lower than values at T0, T2 and T3 in both groups (*p* < 0.05) and that T2 was significantly lower than T3 in EG only (*p* < 0.05). In addition, EG showed greater knee flexion angle values than CG at T3 (*p* < 0.001). No significant effects were found in the healthy leg (*p* > 0.05).

A significant main effect of group was observed in knee angle at heel contact of the healthy leg (F(1,12) = 16.696, *p* < 0.01), with EG displaying greater values than CG at T0 and T2 (*p* < 0.01) (Fig. 3). No significant differences were found in the injured leg (*p* > 0.05).

Table 2 shows the kinetic parameters (Peak_VT_, 1^st^ and 2^nd^ Peak_AP_, Impulse_VT_) at different time measures for the two groups in the healthy and injured leg. ANOVA showed a significant effect of time on Peak_VT_ in the healthy leg (F(3,36) = 3.780, *p* < 0.05) and in the injured leg (F(3,36) = 4.290, *p* < 0.05). However, a significant difference between time measures was only observed in the EG in the healthy leg (F(3,18) = 4.834, *p* < 0.05), with Peak_VT_ being greater at T3 compared to T0 (*p* < 0.05).

**Table 2:** Mean (SD) of normalized kinetic parameters at different time measures for EG and CG groups in the healthy and injured leg. *P* values of significant effect of Time and Group interaction are provided. NBW: Newtons normalized by subject’s body weight. NS: no significant effect of interaction

		**Healthy leg**			**Injured leg**		
		**EG**	**CG**	**TxG**	**EG**	**CG**	**TxG**
**Peak**_**VT**_** (NBW)**	***T0***	1.01 (0.07) ^a^	1.10 (0.10)	NS	1.02 (0.08)	1.09 (0.07)	NS
	***T1***	1.04 (0.04)	1.08 (0.07)		1.04 (0.04)	1.06 (0.07)	
	***T2***	1.03 (0.09)	1.11 (0.07)		1.02 (0.09)	1.10 (0.07)	
	***T3***	1.13 (0.10) ^a^	1.13 (0.06)		1.11 (0.11)	1.13 (0.06)	
**1**^**st**^** Peak**_**AP**_** (NBW)**	***T0***	0.13 (0.03)	0.15 (0.05)	NS	0.11 (0.05) ^a^	0.14 (0.03)	.000
	***T1***	0.13 (0.01)	0.14 (0.04)		0.09 (0.03) ^a^	0.10 (0.03)	
	***T2***	0.14 (0.06)	0.16 (0.02)		0.15 (0.05)	0.12 (0.03)	
	***T3***	0.17 (0.04)	0.15 (0.01)		0.19 (0.03) ^a b^	0.13 (0.02)	
**2**^**nd**^** Peak**_**AP**_** (NBW)**	***T0***	-0.14 (0.03)	-0.15 (0.03)	NS	-0.16 (0.04) ^a^	-0.17 (0.03)	NS
	***T1***	-0.11 (0.03)	-0.13 (0.04)		-0.12 (0.03) ^a^	-0.12 (0.03) ^a^	
	***T2***	-0.15 (0.02)	-0.16 (0.02)		-0.15 (0.03)	-0.15 (0.02) ^a^	
	***T3***	-0.21 (0.04)	-0.17 (0.02)		-0.21 (0.04) ^a^	-0.17 (0.01)	
**Impulse**_**VT**_** (NBW/s)**	***T0***	3.38 (0.79) ^a^	3.41 (2.33)	.024	2.77 (1.33) ^a^	2.93 (1.60)	NS
	***T1***	1.41 (0.41) ^a^	2.41 (1.33)		2.82 (0.99) ^a^	2.65 (0.72)	
	***T2***	3.52 (2.41)	2.30 (0.89)		3.95 (1.69)	3.54 (0.92)	
	***T3***	6.43 (2.17) ^a b^	3.55 (1.61)		5.19 (1.13) ^a^	3.79 (1.72)	

^a^ Significantly different between time measures (*p* < 0.05). For time measure pairing in significant pairwise comparisons, please refer to the text

^b^ Significantly different *vs* CG (*p* < 0.05)

ANOVA showed a significant effect of interaction on the 1^st^ Peak_AP_ in the injured leg (F(3,36) = 8.039,

*p* < 0.001), with no significant effects being found in the healthy leg (Table 2). Specifically, EG displayed greater peak values at T3 with respect to T0 and T1 in the injured leg (*p* < 0.01), whereas pairwise comparisons revealed no significant changes between time measures in CG (*p* > 0.05). Furthermore, EG had greater 1^st^ Peak_AP_ compared to CG at T3 (*p* < 0.005).

A significant effect of time was found in the 2^nd^ Peak_AP_ in the injured leg (F(3,36) = 18.809, *p* < 0.001), with no significant effects being found in the healthy leg (*p* > 0.05) (Table 2). Pairwise comparisons showed that 2^nd^ Peak_AP_ was greater at T3 compared to T0 and T1 in EG (*p* < 0.05), while it was greater at T2 than T1 in CG (*p* < 0.05).

ANOVA showed a significant interaction effect between time and group on the Impulse_VT_ in the healthy leg (F(3,36) = 3.555, *p* < 0.05) (Table 2). In particular, Impulse_VT_ values at T1 were lower than values at T0 and T3 in EG (*p* < 0.05), whereas no significant differences were found in CG (*p* > 0.05). Furthermore, between-group comparisons showed that EG had greater Impulse_VT_ compared to CG at T3 (*p* < 0.05). A significant effect of time on Impulse_VT_ in the injured leg was found (F(3,36) = 6.697, *p* < 0.01), although post hoc analysis revealed that it was significant in EG only (F(3,18) = 7.445, *p* < 0.005) (Table 2),with greater values of Impulse_VT_ being observed at T3 with respect to T0 and T1 (*p* < 0.05).

**Fig. 4:**
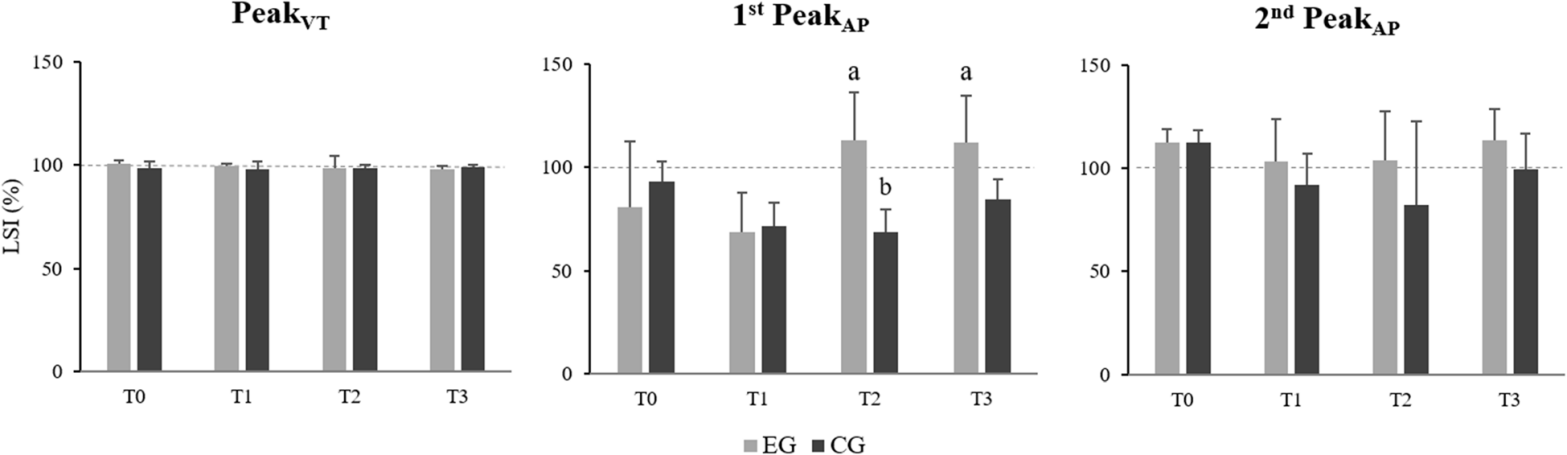
Mean and standard deviation of LSI of Peak_VT_, 1^st^ Peak_AP_ and 2^nd^ Peak_AP_. Horizontal dotted line indicates optimal limb symmetry index value (100%). ^a^ Significantly different *vs* CG (*p* < 0.05). ^b^ Significantly different *vs* T0 (*p* < 0.05)

A significant interaction effect between time and group was observed in LSI of 1^st^ Peak_AP_ (F(3,36) = 6.827, *p* < 0.01) (Fig. 4). Pairwise comparisons revealed that changes between time measures were significant in CG only, with LSI of 1^st^ Peak_AP_ being lower at T2 compared to T0 (*p* < 0.05). Between-group comparisons showed that EG had greater LSI of 1^st^ Peak_AP_ compared to CG at T2 and T3 (*p* < 0.05).

No significant effects were found in the analysis of LSI of MVIC (*p* > 0.05) (Table 3).

**Table 3:** Mean (SD) of LSI of MVIC of knee extensors (Ext_30_ and Ext_90_) and flexors (Flex_90_) at T3 from EG and CG. *P* values of comparisons between groups are provided

	**EG**	**CG**	***p value***
Ext_30_ (%)	62.7 (14.1)	65.9 (22.9)	,751
Ext_90_ (%)	54.8 (24.5)	58.5 (14.5)	,737
Flex_90_ (%)	66.8 (10.4)	67.1 (26.1)	,978

## Discussion

The results of this preliminary study showed that the adoption of the new functional brace improved the kinematics of the injured knee and the management of both anterior–posterior and vertical GRFs during walking at the early stages of the rehabilitation process following ACL reconstruction in comparison to the traditional brace.

In this investigation, the experimental group showed a greater knee flexion angle (about + 10°) of the injured leg as well as a better side-to-side limb symmetry in the 1^st^ peak of antero-posterior GRF (about + 28%) compared to control group during the stance phase of walking two months after surgery. Gait asymmetries have long been identified as a common surgery-related problem in the early rehabilitation after ACL reconstruction [25–27], with possible implications for the initiation of degenerative processes [28]. Subjects showing greater magnitude of gait asymmetries in the first months may present similar imbalances when returning to running [25] and subsequently to sport [26], with walking adaptations detected up to 2 years after surgery [29]. Moreover, it is known that kinematic and kinetic alterations after ACL injury and surgery affect lower limbs bilaterally [30–32], with these alterations also occurring in the uninjured leg during walking activities [16, 17, 26, 33]. This is further confirmed by our results showing significant changes over time in the non-operated leg, especially in the EG (Table 2). Therefore, the early biomechanical improvement in the gait pattern observed in the experimental group may help ACL-R subjects to rapidly restore movement quality and to achieve rehabilitation milestones, thus decreasing the possibility of short and long-term complications [34].

The analysis of GRF during the stance phase of walking showed no difference between groups for Peak_VT_ in the injured leg. On the contrary, the initial vertical impulse showed an increment two months after surgery compared to values before and 15 days after surgery in the EG only. To speculate, the evolution of vertical impulse combined with similar values of Peak_VT_ in the EG could be ascribed to an improved ability in loading the reconstructed limb and in dissipating GRFs over time. This is further supported by the fact that the experimental group has shown greater knee flexion angle, which is known to affect the impact absorption ability [33, 35]. In addition, MVIC analysis two months after surgery showed no significant between-group difference mainly because of the small and heterogenous sample size including ACL-R subjects operated using different autografts (Patellar vs Semitendinosus and Gracilis tendon) which are known to create graft-specific strength deficit in the donor site during the rehabilitation process [36, 37].

The experimental group showed a greater LSI of the first anterior–posterior peak of GRF compared with the control group as early as the first month after surgery and after the adoption of the brace. Furthermore, a reduction in LSI of the first anterior–posterior peak of GRF was observed after one month of brace adoption with respect to pre-surgery level in CG but not in EG. Continuous exercise due to the applied resistance to the hinge of the brace has shown to help in achieving better limb symmetry in the gait pattern compared with traditional post-operative brace locked in full extension and no patient complained of discomfort or pain in the autograft scar area. It has been shown that hamstrings weakness after ACL reconstruction may alter knee mechanics during gait [14]. This creates aberrant adaptations, shifting cartilage loading and therefore contributing to the development of joint degenerative process [38–40]. In addition, the presence of hamstrings weakness after surgery seems to be related to the kind of rehabilitation undertaken by the patients [41], thus, early post-operative approaches aiming at targeting hamstrings strengthening and restoring normal gait from the first phases may be helpful in the long-term morphofunctional recovery perspective. We have limited our evaluations to the first 2 months after ACL surgery, in order to rule out other factors underlying functional recovery such as any change in daily living activities, adherence to the rehabilitation protocol and personal motivations. However, the small sample size in this study makes difficult to extend the current findings to general population and, therefore, should be taken into account when applying such evidence to clinical practice.

The study presents limitations that need to be considered when interpreting the current findings. The small sample size may have influenced the observed results as an effect of inter-subject variability is likely expected with a low number of participants, thereby making the study preliminary. For instance, the different knee angle at heel contact between the two groups that was observed in the healthy leg before surgery (T0) could be attributable to the low number of included patients. In addition, the presence of different types of graft (PT and ST-GR), although almost balanced across the two groups, may have played a role in the observed effects, especially MVIC measurements, as it has been shown to influence the strength of knee flexor and extensor muscles [42]. For future perspective, the inclusion of a greater number of patients, possibly with similar characteristics (i.e. same type of graft), would therefore decrease the heterogeneity of the sample, likely strengthening the present findings and unveiling more subtle differences related to the adoption of the proposed functional knee brace.

In conclusion, the present preliminary results indicate that, for ACL reconstructed subjects involved in the early post-surgery rehabilitation, the adoption of the new functional brace with continuous leg-applied resistance to knee flexion allows a greater recovery of injured knee kinematics and better management of sagittal ground reaction force during gait, particularly at weight acceptance, compared to traditional knee brace locked in full extension. To date, the medical choice on whether to use a post-operative brace is up to the surgeons’ discretion due to its non-significant additional benefit on the knee function and stability in the short term when added to standard therapy [9–11]. However, preliminary findings from this study suggest that the adoption of the new functional knee brace could be considered as a viable alternative to traditional brace for a better recovery of the gait biomechanics, reducing the risk of joint damaging while adding an intrinsic rehabilitation with the persistent applied resistance. Noteworthy, this could be considered to be adopted in a selected population which is not able to follow a structured and supervised rehabilitation practice during the early stages of the rehabilitation process. Nevertheless, future studies with larger and more homogeneous samples are needed to provide more robust evidence and, in addition, to understand whether the use of this type of braces can help the recovery of strength after ACL reconstruction.

## Data Availability

The datasets used and/or analysed during the current study are available from the corresponding author on reasonable request.

## Abbreviations

ACL: Anterior cruciate ligament
ANOVA: Analysis of variance
AP: Anteroposterior
GRF: Ground reaction force
LSI: Limb symmetry index
MVIC: Maximal voluntary isometric contraction
PT: Patellar tendon
ST-GR: Semitendinosus and gracilis
VT: Vertical
